# The Benefits and Challenges of the Parental Monitoring of YouTube in Adolescents’ Lives: A Qualitative Study of Emotion and Sleep Regulation

**DOI:** 10.3390/bs15060805

**Published:** 2025-06-12

**Authors:** Linda Charmaraman, Ramona Smucker, Sally A. Theran, Srimayee Dam, Jessica Anthony

**Affiliations:** 1 Youth, Media, & Wellbeing Lab, Wellesley Centers for Women, Wellesley College, Wellesley, MA 02481, USA; rsmucker@wellesley.edu (R.S.); sd3837@tc.columbia.edu (S.D.);; 2 Department of Psychology, Wellesley College, Wellesley, MA 02481, USA; stheran@wellesley.edu

**Keywords:** YouTube, social media, adolescent development, sleep health, emotional regulation, parental monitoring

## Abstract

YouTube is the most popular social media platform for children and adolescents, yet relatively little research has been conducted on adolescents’ use and their motivations for use. Prior research has predominantly focused on measuring the quantity of time spent on the platform, and less is known about the motivations and self-awareness of using YouTube as children turn into tweens and teens. Stemming from a larger survey study of adolescent social technology use, we interviewed a subset of 35 youths (50% female, 49% male, 1% non-binary) to qualitatively explore the benefits and challenges of YouTube use. Thematic analyses revealed the wide range of emotional responses and regulation that were attributed to YouTube use, including humor, fear, anger, insecurity, and anxiety. Some participants experienced wishful identification with YouTube influencers, and others viewed YouTube as entertainment or an escape from boredom. Sleep regulation was mixed, in that for some, YouTube was a distraction from getting enough sleep, and for others, it helped them fall asleep. Parental monitoring was a developmental challenge as the youths described their parents as lacking knowledge about the length of time they spend on the platform and/or the risky YouTube content that they watch, particularly as they got older. These exploratory findings may be pertinent for parents, educators, and clinicians.

## 1. Introduction

According to a recent nationwide poll from the Pew Research Center, YouTube continues to be the most popular social media (SM) platform among teens, with 95% of teens having used this site (Vogels & Gelles-Watnick, 2023). Three-quarters of teens (77%) report using YouTube every day. Through YouTube, teens learn about a wide variety of informational topics and find games and entertainment; hence, it provides a sense of community and occupies a vital role in the media life of teens (Pires et al., 2021). In a recent survey, which involved participants between the ages of 6 and 17, over half of 1000 children and teenagers wanted to be a YouTuber or vlogger when they grew up (Yasaroglu & Boylu, 2020). One study specifically stated that more and more tweens aspired to become YouTubers as the influencers they followed were famous and seemed financially stable (Melendres, 2019). Despite YouTube’s massive popularity compared to the next most-frequented sites, such as TikTok and Instagram, there is relatively little research conducted on how YouTube affects tween and teen users. The goals of the current exploratory qualitative study are to understand early adolescent motivations to use YouTube, any positive (e.g., emotion regulation) or negative (e.g., poor sleep) consequences of YouTube consumption, and parents’ awareness of YouTube’s impact on their children.

### 1.1. What Motivates Youth to Watch YouTube?

As adolescents’ motivations for using internet sites have important educational and emotional implications, it is critical to understand how they relate to content on sites such as YouTube. The use of YouTube videos in the classroom has resulted in stimulating students’ interests, engaging students’ attention, fostering creativity, increasing collaboration, and improving retention and overall academic performance (Ogirima et al., 2021). Parental attitudes are equally important in their child’s YouTube use. A majority of parents (about 94%) either loved or liked YouTube, and almost 70% used YouTube on a daily basis. In addition, 74% of children reported that their parents allowed them to watch YouTube, and 43% of children between the ages of 6 and 12 often watched YouTube with their families (Izci et al., 2019).

Previous studies related to televised content have offered a theoretical framework for motivations for following certain shows or channels. The way that adolescents relate to media personalities has been theorized as a form of wishful identification (Feilitzen & Linné, 1975; Hoffner & Buchanan, 2005), in which media viewers strive to emulate certain characters, and, at times, as a type of parasocial relationship (Horton & Wohl, 1956; Rubin & McHugh, 1987): a one-way, imagined intimate relationship that viewers may develop with their favorite celebrity. In particular, one study noted that adolescents highlighted favorably salient media figures, such as actors and musicians, for parasocial attention (Gleason et al., 2017). This parasocial relationship framework has been extended to the YouTube sphere, with scholars theorizing that these relationships mimic those created by traditional mass media (Kurtin et al., 2018; Rasmussen, 2018). Balleys et al. (2020) noted that several studies on adolescents aged 12–19 revealed a twofold social recognition process that incorporates a capacity to recognize oneself in others—such as figures with whom they can identify—and a need to be recognized by others as beings of value. Tolbert and Drogos (2019) identified the time spent watching a YouTuber as being positively related to both the strength of a tween’s (aged 9–12) wishful identification and their parasocial relationship with that YouTuber.

Given that teen boys are more likely than girls to use YouTube (Vogels & Gelles-Watnick, 2023), studies are emerging that demonstrate gender differences in YouTube viewing. Most tweens favored YouTubers of the same gender as them and who they perceived as funny, and boys displayed stronger wishful identification with YouTubers whose content had increased violence. Boys and girls also formed parasocial relationships based on different attributes, with boys reporting stronger parasocial relationships with YouTubers they perceived as successful and attractive, and girls gravitating towards YouTubers seen as being well liked. These findings suggest nuanced reasons that young YouTube users seek out role models or imagined friendships with online content creators, based on gender. Tolbert and Drogos (2019) explained these differences by applying gender schema theory (Bem, 1981) and suggested that one of the attractions of the YouTubers watched by various genders is their role modeling, namely their teaching and modeling for adolescents how to “be” their gender.

Media consumption and interaction may also be analyzed through a uses and gratifications framework (Katz et al., 1973), which argues that media viewers make active choices about what they consume in order to meet their social and psychological needs, for instance, emotional regulation. In applying this theory to newer forms of user-generated media, Shao (2009) noted that YouTube users have different motivations for engagement on the platform, including consuming content to learn and entertain and interacting with content to find social connection. Through the use of YouTube, college students are known to satisfy their cognitive needs, as well as gratify their personal, affective, and social integrative needs (Pasha et al., 2021). Platforms like YouTube also demand minimal effort (e.g., easily accessible content) from users while granting the maximum control of what they view, creating a pattern of use that is distinct from engagement with traditional media. Creating an account allows users to engage with content more actively (e.g., by subscribing to individual channels, online spaces that host video content from a single creator). Although YouTube has historically been considered a site for passively watching and sharing videos, YouTube users can also interact with one another by liking, disliking, and commenting on videos, which can create a sense of community, encourage discussion, and even promote the credibility of the content posted (Khan, 2017). Regarding content consumption on YouTube, gender was also another factor that predicted participatory behaviors. The results show that being male is predictive of reading comments but not of viewing videos. A potential reason is that females may be inclined to avoid reading and writing comments on YouTube.

### 1.2. Rise of YouTubers and Their Influence on Youth

While YouTube can be an educational resource (e.g., DIY projects) and a source of entertainment, there are potential risks for individuals under 18 using the platform. For instance, a study conducted by the Pew Research Center states that 61% of parents surveyed said their child has seen content on YouTube that they felt was unsuitable for children under the age of 11 (Smith et al., 2018). Parents may notice that their kids see creators like Tana Mongeau and Jake Paul, who built their online fan bases as teenagers, live extravagant lifestyles by being “authentically themselves” (Tenbarge, 2019). Yet, Jake Paul, his older brother Logan, and Mongeau are more recognizable to adults for their frequent scandals, rather than their personalities. Some of these controversies include Logan Paul filming a dead body in Japan’s suicide forest video and his younger brother turning his Los Angeles neighborhood into a “living hell” for his neighbors (Tenbarge, 2019). However, much of the parental concern over risky content traces back to how youths can easily access this platform, possibly without their parents’permission. When one large scale study asked children between the ages of 6 and 17 what they wanted to be when they grew up, 75 percent said they wanted to be a YouTuber or a vlogger (Dirnhuber, 2017). However, recent scholarship indicates that creating original YouTube content is less common than other uses among teenagers, and that content creation is mostly dominated by male adolescents who upload videos focused on gameplay, as opposed to attempting to become famous as a YouTuber (Pires et al., 2021). Instead, YouTube more commonly functions for teenagers as a music player, an arena for accessing media and educational information, and a social platform (Pires et al., 2021). More research is needed to understand the extent to which adolescents are cognizant of actively or passively consuming YouTube marketing messages and autoplay features.

### 1.3. Sleep Patterns and Social Media Use

Another area of concern related to adolescent self-awareness is in their self-regulation around YouTube viewing, which may affect sleep patterns. Prior research on adolescents (aged 13–15) has demonstrated that heavier social media use is correlated with insufficient sleep (Scott et al., 2019). Research examining the specific influence of YouTube on adolescent sleep is more limited. A survey study conducted with 11–15-year-olds revealed that watching YouTube videos before sleep was correlated with a later bedtime and reduced hours of sleep (Charmaraman et al., 2021). The quantity of social technology use (e.g., checking social media, problematic internet behaviors, mobile use), the content viewed (e.g., emotional or violent videos, risky behaviors), and the social context (e.g., bedtime behaviors, starting social media at an early age) were also significantly related to later bedtimes and fewer hours of sleep on school nights. Pillion et al. (2022) investigated the associations among adolescents’ (aged 12–18) evening use of technology devices and apps, night-time sleep, and daytime sleepiness, and found thatwatching YouTube and using gaming consoles were associated with greater odds of insufficient sleep. YouTube was associated with increased odds of sleeping less than 7 h on school nights. Although there are concerns around the quantity of time spent on YouTube before bedtime, which may affect sleep quality and presumably academic functioning, more studies are needed to understand the motivations behind this use at night, which may help adults tailor their guidance.

### 1.4. Parent Mediation of Media and YouTube Use in Early Adolescence

Parent involvement can be protective in some way to buffer any negative effects of media consumption (Padilla-Walker et al., 2018). Prior research has focused on the parental mediation of children’s YouTube behaviors, though families of adolescents who are beginning to consume media without parental supervision are also a new research target, at least partially due to new access to smartphones and mobile devices (Rideout & Robb, 2019), and because parents of adolescents feel less able to mediate their adolescents’ device use (Charmaraman & Grossman, 2014). Existing research has examined a variety of parental approaches to monitoring and understanding children’s consumption of video content; among these, the most popular are active mediation, restrictive mediation, and co-viewing. Restrictive mediation, in which parents set rules limiting their children’s media use, can be effective at reducing the amount of time children spend watching online videos; however, restrictive rules are less effective at protecting children from risks than active mediation, in which parents engage their children in discussions around the content they watch, or co-viewing, in which parents watch media with their children (Chen & Shi, 2018; Steinfeld, 2021). Both of these latter strategies grant youth greater autonomy and instill in them the skills to navigate content on their own terms as they grow older (Steinfeld, 2021). Alternatively, youth who are subject to restrictive mediation often compensate for the restrictions they face by watching riskier content when possible and are at a higher risk for media addiction (Chen & Shi, 2018). Although parental mediation strategies for YouTube use have largely gone unexplored, tweens have reported low levels of co-viewing YouTube videos with their parents (Tolbert & Drogos, 2019), indicating that this strategy may be less developmentally appropriate given growing needs for autonomy and also less practicable when adolescents are primarily consuming video content on mobile devices.

Currently, adolescents are required to navigate digital challenges and risks that differ from those faced by their parents/teachers. A qualitative interview study of 9–12-year-olds from New Zealand (McDonald-Brown et al., 2017) queried how effective teachers and parents can be in taking a supportive role in developing internet self-efficacy among their children and students. There was a range of parental approaches for internet use, from highly involved to barely involved (e.g., no restrictions). Most of the participants reported the existence of rules, although some claimed not to have any. The responses also indicate that children generally feel positive about communicating with their parents. Yet, many of the participants claimed to have a higher level of knowledge about the internet than their parents, primarily due to technical knowledge. The results indicate that the role of a parent/caregiver has expanded significantly to include the vast range of issues that the digital frontier brings with it. Parents and teachers have been taking a supportive role in developing digital citizenship skills and increasing self-efficacy among children and students and the way they face challenges when online. More studies are needed to explore how adolescents perceive the role of parents in mediating their YouTube use.

### 1.5. Current Study

Early adolescence is a critical time period for which we aim to understand transitions in YouTube use, as the content adolescents access shifts in its maturity level, and adolescents may test out new levels of autonomy from their parent’s knowledge as they age. Utilizing the uses and gratifications framework (Katz et al., 1973) for studying why and how people use media, the following qualitative research questions focus on how middle school students use YouTube to gratify their needs (e.g., entertainment, identification, emotion regulation), explore parental knowledge and mediation of YouTube use in early adolescence, and any positive and negative consequences of YouTube use. Additionally, we explore how YouTube influencers may operate as a target of wishful identification.

### 1.6. Research Questions

- What motivates early adolescents to watch YouTube channels? Are there any gender differences in its appeal?
- What benefits and challenges relate to YouTube consumption in adolescence, particularly regarding emotion regulation and sleep?
- How much do parents know about and mediate their adolescents’ YouTube consumption?

## 2. Materials and Methods

### 2.1. Participants and Procedures

We recruited schools in the Northeast US (3 public, 1 private) of varying sizes and demographics, reflecting urban and suburban areas, to participate in a digital media study in 2018–2019, as a part of a larger longitudinal study of adolescent social technology use (Charmaraman et al., 2020, 2021). We analyzed data from wave 1 of this ongoing data set, collected in 2019, using Qualtrics software. After obtaining IRB approvals to conduct the study using a waiver of consent document, we distributed parental informed consent/opt-out forms through parent email listservs and school newsletters. The schools and programs provided the participants with Chromebooks during a pre-scheduled period to take the survey. Trained research team members proctored the web-based surveys in person, supervising the assent process, and were available to answer questions. The participants were told that their answers would be kept confidential and that they did not have to answer any questions they found uncomfortable. The surveys were available in English, Spanish, and Portuguese. The students were entered into a raffle prize draw to win gift cards, and embossed pens were given to every student during the survey’s administration. The schools were provided with a small honorarium for providingaccess to their students. The participation rate was 85% across all the schools and programs.

The survey participants were 50% female, 49% male, and 1% non-binary gender or missing, and they represented diverse racial/ethnic backgrounds (46% White, 13% Latinx, 10% Black, 7% Asian, 7% biracial, 5% Native American, <1% Middle Eastern, and 12% other/unknown race/ethnicity). The mothers’ average level of education was 3.79 (SD = 1.12), equivalent to having completed some college or completed college. 23% of the participants received free or reduced-price lunches. Of the students, 73% reported living in a two-parent household, and 50% reported living in a home with an older sibling. The average age was 12.69 years (SD = 1.21).

The survey participants were invited to a follow-up interview about their social technology use. Out of the students who volunteered, we selected students who had varying levels of YouTube use and parental mediation of their technology use. Once selected, we contacted their parents/guardians for active consent, and the students provided verbal assent to participate before each interview. We interviewed 35 (20 girls, 15 boys) middle school students. Our sample was slightly younger than the larger survey sample, with sixteen 6th-graders, ten 7th-graders, and nine 8th-graders. The interview sample was also less ethnically diverse than the larger survey sample, with 24 White, 5 Latinx, 3 Asian/multiracial Asian, and 3 Middle Eastern participants.

### 2.2. Qualitative Analysis

One author conducted primary open coding using NVivo 11.0 (QSR International software, Burlington, MA, USA) until all the relevant quotes were bucketed and coded. Themes and subthemes were discussed with a second author to clarify the emerging codebook and conduct a secondary coding process (Strauss & Corbin, 1998) related to the quantitative findings from the larger survey study. When possible, we identified any YouTube channels that were most popular amongst our sample within our quoted examples in order to put the quantitative results into context. Table 1 below is a joint display of our results by YouTube channel. All names reported in this study are pseudonyms.

## 3. Results

We identified three primary themes related to the larger mixed-method study that were most relevant to YouTube use: (1) motivations, appeal, and gratifications of YouTube, (2) self-regulation and sleep, and (3) parental knowledge and restrictions of YouTube use.

### 3.1. Motivations, Appeal, and Gratifications of YouTube

#### 3.1.1. Motivation to Regulate Emotions

The participants described a range of emotions when watching YouTube, both positive and negative. On the positive end, about two-thirds of our interview sample reported that they gravitated towards their favorite channels because of humorous or entertaining content. Some of the participants describe how the unpredictability of the content was humorous to them. For instance, Lauren, an eighth-grader (age 13), describes how some of her favorite channels, like James Charles and David Dobrik (both channels are considered high-risk), make her “laugh a lot” because they are not “normal”.

Similarly, seventh-grader Nicole (age 12) describes the emotional nature of a gaming channel named Jacksepticeye, which often showcases the high-stakes nature of winning and losing: “He makes a ton of jokes every so often, and sometimes he gets mad at it, and it’s just funny watching people get mad (‘raging’) over a game”. Jason, a sixth-grader (age 11), enjoys one channel where the YouTuber would “get super mad” because “every time he dies in the game, he smashes his keyboard on the table”, which Jason finds “wicked funny”. Some of our participants reported using YouTube to help them regulate their emotions, such as Carlos, a 13-year-old eighth-grader, who uses YouTube to “to calm down from an argument with [his] parents” because “when [he is] annoyed or sad…the funny just makes [him] forget about what happened”. One participant, Dimitri, a 13-year-old seventh-grader, reports using YouTube as part of her healing or recovery process post-surgery: “It was a way to relax”.

In contrast, some of the participants reported negative emotions while watching YouTube, such as Lizzie (a 13-year-old eighth-grader), who described how watching YouTube sometimes makes her “just feel insecure” because she “see[s] all these perfect people”. She also struggles with anxiety about Momo, a viral internet sensation that was rumored to encourage children to engage in self-harm and suicide, and she describes often being “kind of scared when [she] get[s] home… because of it”. Carlos, an eighth-grader aged 13, also expresses discomfort about Momo, who he characterizes as “a challenge that persuades some teenagers around my age to commit suicide”: “Personally I’m kind of scared of like that [Momo’s] face”. Lauren (an eighth-grader, aged 13) notes that sometimes she feels “angry” when “a celebrity says something that’s rude”. However, she thinks that most of the things she watches “uplift” her and do “not make [her] feel sad”. At times, the participants used YouTube as “background noise” while doing homework (e.g., eighth-grader Teresa). Brittany J. (an eighth-grader) described her avoidance of the negative emotions related to boredom, and used YouTube as a multitasking outlet:

Normally, I can’t just watch something. I’ll do something else. So I’ll do my homework while I’m watching it. It’s kind of—they’re entertaining, but also some parts get a little boring, so I’ll do my work during those parts.

(Brittany J.)

#### 3.1.2. Wishful Identification

In terms of identifying with YouTubers, many of the interview participants described their favorites as “relatable” or as people they wished to emulate, consistent with theories of parasocial relationships and wishful identification. Seventh-grader Catherine watches Emma Chamberlain, described as being “really funny and she doesn’t really care, like she’ll just go out with no makeup and a messy bun, and her videos are so relatable, and she is really funny”. Linda (a 12-year-old sixth-grader) said, “I’d say that they’re funny, and relatable, because they might have the same concerns as you…Or you could learn something, where you could get something, what you could do to fix a problem or something”.

While many of the participants expressed admiration for and a wish to emulate the YouTubers they watch, the reason for this admiration tended to differ for the male and female participants. The boys in our study most often described gaming skills as the source of their admiration. Jack, aged 12 and in the seventh grade, finds watching people playing Fortnite, a popular video game, “entertaining to watch” because “the people that are playing are really good at it”. Other boys admired the financial status of their favorite YouTubers as the source of their admiration; Carlos, an eighth-grader, describes how he “want[s] to see how their life is being rich…like what places they go, cause sometimes they go on vacation to beautiful places that [he] want[s] to go”.

In contrast, the girls in our study more often described how they tended to aspire to the lifestyle or the unique identities of their favorite YouTubers. For some, this admiration is inspiring; Jenna, in the sixth grade, likes watching “people who are really positive on their channel”. In particular, she finds the Ace family admirable because “they lost their brother because of his heart…They always talk about him like a prayer. They say positive things that their brother said”. Catherine, in the sixth grade, watches DIY channels because “I just think what they make is really cool, and they’re like, they seem to be very nice people”. However, this admiration can also be problematic. Lizzie, who is in the eighth grade, enjoys “looking at all the nice stuff they [YouTubers] have, their homes, like all the places they can go”, but she describes how watching these channels sometimes cause her to “just feel insecure” because she “see[s] all these like perfect people”. Another respondent, Catherine, in the seventh grade, watches many videos about “how to be skinnier or workouts to do” and “how to get a bikini body or how to get a flat stomach or stronger arms”. She believes these videos “make [her] better” because they inspire her to “improve” her body, though she describes struggling to stay “motivated” to do so. Thus, for some of our participants, particularly girls, the admiration and emulation of YouTubers appear to be linked to insecurities and/or a problematic body image.

### 3.2. Self-Regulation and Sleep

Self-regulation was a common struggle for our younger students, particularly at bedtime. George, a sixth-grader, reported that his parents knew about the content he watched (e.g., games and sports), but they imposed a curfew at around 9–9:30 p.m. He revealed that it was hard to self-regulate his YouTube at night: “Sometimes what I watch is interesting—like a new game came out—and I didn’t get to play it, and I watch something about it. See that wouldn’t make me sleep”. Another sixth-grader, James, also notes that sometimes he finds it hard to sleep because “it’s entertaining and he can’t stop watching videos”. Tyler, also in the sixth grade, sometimes finds it “hard to transition” from watching “YouTube to going to sleep…so quickly”.

Our interviewees who use YouTube right before bedtime report mixed effects of YouTube use on their sleep patterns. Several of the participants noted that they used YouTube right before bedtime but had no issues falling asleep afterwards. For example, Chad H. reported that he had no problem stopping his YouTube use before sleeping because he knew he was “ready to go to bed” when he was “getting tired”. Several of the participants even said that YouTube use was helpful to them when trying to fall asleep. Bob (a seventh-grader, female-identifying) uses YouTube if she can’t sleep—“If I can’t sleep, I just kinda watch something and then I go to bed”. However, Bob also said that she does not have a problem with putting away her phone and quitting YouTube and other social media because she “really like[s] sleeping”. Catherine (a seventh-grader) reported that she used YouTube “right before I’m about to go to bed because for some reason it actually helps me fall asleep… Like if I don’t use it, I won’t fall asleep until like 1:00 a.m., but if I do use it, I’ll fall asleep at 10:30 or whenever I put my phone down”. She did admit that “sometimes if I’m watching a really funny YouTuber, I might stay up a little later than I want to”, but that she usually goes to bed immediately when she finds out the time. Gabrielle (a rising-ninth-grade 14-year-old) mentioned that her “mom makes me put it out in the hallway, ‘cause if it dings or anything, it could wake me up, so she doesn’t like it to be in my room”. She also said that she “used to sneak out and look at her phone but not anymore”.

Other participants struggle more to sleep after watching YouTube at night. Lauren, who sometimes found it difficult to put the phone away at night if she was watching “videos I wanna watch or my favorite show”, described it being difficult to sleep after watching certain YouTube videos, such as Shane Dawson, because “sometimes I’ll be scared to go to sleep after, just like if he does the conspiracy type theories”. Other participants describe finding it difficult to put away their devices before bedtime when they have been watching YouTube. For example, Jack (a seventh-grader) delays his bedtime because of YouTube “cause it’s entertaining and I like watching it”.

### 3.3. Parental Knowledge and Restrictions of YouTube Use

Our youngest interview participants tended to report at least some parental restrictions on the YouTube content they could watch and the length of time they could watch or having night-time curfews. Sixth-grader Alex said that his parents have a rule of “no bad words in the content” but notes that he does not encounter YouTube content with that problem. James, a sixth-grader, mentioned that his parents were aware of the content he watches and that his parents will sometimes tell him to go to bed “if I have been on it for too long”. The participants reported that their parents had installed safeguards such as passwords before downloading a new app, even though in at least one case, there was a two-way level of trust:

Well, I ask them every time like I’m getting an app, cause parental controls, you have to ask them and then put in the password. I know the password, but I still put it in anyway. I ask them. And they get a notification when I download an app anyway.

(Bob P., a sixth-grader)

In some families, there was ongoing dialogue about what was appropriate content. Jenna, a sixth-grader, demonstrated how she and her parents frequently checked in about content consumed on such channels as the Ace Family:

If I’m on YouTube, I’ll usually tell her what I’m watching and every time I’m on YouTube, she’ll just say, ‘What are you watching?’…If I find something on YouTube that’s bad, I usually tell them. I make sure that I can’t get to that…They’re sure that I’m not on bad things so that’s why they let me use it. They tell me what not to do. I don’t watch anything bad.”

(Jenna)

Other participants described specific content that parents did not want them to be exposed to, such as “kissing and all gross stuff” (Victoria, a sixth-grader) or risky behaviors in older youth (e.g., vaping, profanity, or messages of self-harm or harming others). Distant, passive forms of YouTube supervision were also reported, such as the case of Jason (a sixth-grader): “They know who I watch and-because I usually have my volume up really high, I don’t know why, and they just listen to it, and they know what I’m watching”.

Parental rules around YouTube limit-setting often revolved around whether it was going to get in the way of schoolwork, such as making sure homework and chores are completed before logging onto YouTube. Examples of rules or curfews related to encouraging a positive educational context include the following: “Can’t use the tablet in the morning. Only allowed after dinner if homework is done” (Jessica, a sixth-grader). “I have gotten my phone disabled once though. My mom can disable it whenever if I’m not doing homework” (Joe, a seventh-grader). “Do your homework before, and it shouldn’t affect how well you do in school” (Chad H., an eighth-grader).

The majority of the interview participants who reported either low or no parental knowledge of or involvement in restricting their YouTube use were in the older grades. The participants shared how their technology rules used to be stricter, but then their parents relaxed them at some point, often not knowing the reason why:

So there’s a thing called “screen time” but you can set limits on how long, and I can set limits on my phone, but since her phone controls mine, she would put an hour limit on each social media app, and then… we went on a vacation and we had a lot of long car rides, and she turned it off for that and I guess she never turned it back on, I guess.

(Samantha, an eighth-grader)

A common theme was that teens felt their parents trusted their judgment regarding their YouTube behaviors. Andrew, a seventh-grader who watches Dude Perfect, along with various gaming channels, says that his parents do not have any child safety protections on his computers: “They don’t know everything I see. They know I don’t do anything bad on YouTube”. Brittany Jones, in the eighth grade, says that her parents do not have any specific rules about YouTube because “My parents trust me to know what’s appropriate and what’s not. And if there’s something that isn’t appropriate, to stay away from it or don’t do it or repeat”. Brittany Jones is left to self-regulate her own YouTube use, converse to the case of sixth-grader Jenna above, who relied on her parents to help her determine what was age-appropriate. Catherine says that she also does not have any rules from parents about YouTube content because “my mom kind of just trusts me, so I’m not really that type of person to watch anything bad I guess” but notes that some of the channels she watches have some inappropriate content: “David Dobrik—he isn’t the best role model…they all do swear, which I don’t really like. And it’s kind of annoying”.

In the no-monitoring category, the participants described parents as being somewhat knowledgeable about the content but not having any restrictions on what they can and cannot watch: Lizzie, in the eighth grade, says, “I really haven’t talked to my parents with that kind of stuff…I don’t really think it really matters to my parents”. Tessa, an eighth-grader, reported that she and her sister “often stay in their room and put YouTube on the TV and on their cell phones”. Some of the participants revealed that their parents think they watch less risky content than they really do. Nicole, in the seventh grade, who watches the gaming channel Jacksepticeye, said that her parents are not usually aware that she watches that channel: “Usually whenever she catches me, I’m usually watching animal or food videos”.

## 4. Discussion

The current study is an in-depth qualitative exploration of the benefits and challenges of YouTube use in the early adolescent years with a focus on adolescents’ awareness of the role YouTube plays in their emotional regulation, sleep, and parents’ awareness of their children’s YouTube activities. While previous findings about the motivations of YouTube use (e.g., Gleason et al., 2017) have been limited in age range and were mainly international-based, our current U.S.-based study was conducted on an overlooked early adolescent period of development in YouTube use. Prior research with tween YouTube users has also found that dispositional, developmental, and social factors predict YouTube habits (Taylor & Cingel, 2023). The existing literature highlights theories pertaining to why children and adolescents emulate and follow popular personalities, primarily on traditional mass media (Kurtin et al., 2018; Rasmussen, 2018; Tolbert & Drogos, 2019) and less often on social media, such as YouTube influencers. Extending past work on the social and emotional regulation contexts of youth using YouTube (Katz et al., 1973; Khan, 2017; Pasha et al., 2021; Shao, 2009), the current exploratory study found that adolescents experienced both positive and negative emotions when engaging with YouTubers online. About two-thirds stated that they continued to watch their favorite channels because the content was humorous and entertaining. Others noted portrayals of extreme emotions helped regulate or balance-out their emotions and allowed them to escape boredom. Additionally, some felt fear, anger, insecurity, or anxiety while watching content related to self-harm and teenage suicide. These findings highlight the self-awareness of emotions that play a role in adolescents’ motivations for using YouTube, such as gratifying their emotional needs and, in some cases, their need to identify with an influencer who they find appealing and relatable. This gratification of emotional needs can pose challenges for parental monitoring since guidance around setting limits for YouTube consumption is not a one-size-fits-all mandate. At any given time, a youth can experience a range of both positive and negative emotions while using YouTube, thus it may be important for parents to understand the motivations around YouTube use at different ages and stages by having ongoing dialogue with their child before setting regulations to control its use.

Unlike past research (e.g., Tolbert & Drogos, 2019), the current study specifically focused on the difference in interests among boys and girls regarding admiration for YouTubers. The boys described the “gaming” skills of the YouTubers as being really engaging for them to watch. On the other hand, the girls sought the lifestyle or unique qualities of their favorite YouTubers. Nonetheless, it was found that comparison with YouTubers’ “perfect and aspirational lives” could result in insecurities among teen followers, irrespective of gender. More research is needed on the parasocial relationships that young people engage in while on YouTube in the adolescence period (Kurtin et al., 2018; Rasmussen, 2018).

Notably, students reported varying perspectives on YouTube use in relation to their sleeping patterns. Numerous participants remarked that they use YouTube prior to bedtime but have no difficulties falling asleep afterwards, and others felt motivated to continue watching because they were being entertained by their favorite influencer. Most of the participants could identify their own sleep patterns, but there was little effort to change any negative patterns of viewing YouTube that may reduce sleep. In prior studies, some adolescents between the ages of 12 and 18 noted that social media use on other apps can be neutral or even beneficial when it comes to falling asleep on school nights (Pillion et al., 2022). These other social technologies included phone calls, text messaging, Instagram, Facebook, Snapchat, Twitter, Reddit, Tumblr, Spotify, Netflix, Viber/WhatsApp, gaming apps, audio books, and “other”. On the contrary, some of the participants within that same age range found it tough to put away their devices before bedtime whenever they were on media platforms such as Netflix and YouTube. Prior research assessing the effects of YouTube on adolescents’ sleeping patterns has shown that watching YouTube videos before sleep was correlated with a later bedtime and reduced hours of sleep (Charmaraman et al., 2021). These particular findings are consistent with the idea that social media use can displace sleep (Scott et al., 2019; Pillion et al., 2022). A recent study has found that poor sleep quality influences feelings of social exclusion, self-control abilities, and emotional regulation (Wang et al., 2024). The inter-relationship between sleep and emotional regulation underscores the need for parental monitoring to enhance both of these developmental tasks.

Some of the participants reported that their parents are aware of the content they watch, which supports past research on how communication can be effective for families regarding their daily internet use (McDonald-Brown et al., 2017). However, some adolescents endorse that their parents believe they watch less risky content than they actually do, unveiling some potential blind spots for the parental mediation of YouTube content. Past research has shown that youths who are subject to *restrictive mediation* often compensate for the restrictions they face by watching riskier content when possible and are at a greater risk for media addiction (Chen & Shi, 2018). On the contrary, different types of mediation, such as *active mediation* or *co-viewing,* grant youths greater autonomy and instill in them the skills to navigate content on their own terms as they grow older (Steinfeld, 2021).

### Limitations and Future Research Directions

The current study had a number of limitations. First, the data collection occurred during a pre-COVID-19 pandemic period, thus the exact YouTube content will not mirror what adolescents currently watch (e.g., the ACE family channel is no longer active due to divorce but still has over 18 million followers as of 2025). Although algorithmic content suggestions on YouTube are in constant flux, the current study explored adolescents’ favorite channels that are regularly subscribed to, which is more indicative of their consumption patterns, rather than random channels they might encounter due to platform suggestions. Nevertheless, the themes explored in the current study underlying adolescent motivations, coping strategies, and parental mediation may continue to have relevance in a post-pandemic world. Second, as described in the Materials and Methods Section, the adolescents who participated in the qualitative study were not necessarily representative of the larger study. Third, due to the nature of this qualitative inquiry, it is impossible to determine causation in terms of watching YouTube and outcomes such as distractibility or sleep disturbances. Fourth, given the open-ended nature of the questions in the qualitative interview, it is challenging to understand the role of certain psychological phenomena, like parasocial relationships. The adolescents interviewed are at a developmental stage where they may not necessarily expand on their responses. Given that without specific probing about the degree of wishful identification with YouTubers, future research could focus on more direct questioning about parasocial relationships, expanding upon research that demonstrates a connection between YouTube viewership, the importance of relationships, and parasocial relationships (Kurtin et al., 2018). Future research using mixed methods research could focus specifically on the relationship between gender and the function/utility of YouTube in adolescents’ lives, including the gender of the parent monitoring the usage. The possibility that YouTube videos can be used as a beneficial sleeping aid also merits further investigation. Finally, to our knowledge, there is a dearth of both quantitative and qualitative research on parents’ perspectives on the role of YouTube in their children’s lives.

## 5. Conclusions

The goal of the current qualitative study conducted during a pre-COVID pandemic period was to explore how adolescents use YouTube for gratification, the role of parental monitoring, and any benefits and consequences related to YouTube use. The key findings indicated that the respondents reported both positive and negative emotions related to YouTube, which poses challenges for parents to provide guidance to their children that may depend on their children’s underlying motivations and ability to self-regulate. Female respondents were more likely than male respondents to report wanting to be “like” the person they watched, whereas male respondents were more likely than females to report gaining skills from watching their favorite YouTuber. For some respondents, YouTube interfered with sleep, whereas it may have helped with sleep for others. Future mixed-method and multiple-reporter research is necessary to understand the interaction between parental perspectives on YouTube videos and their teens’ use of the medium.

The current study highlights the appeal of following YouTube channels not only for the content but to observe emotions in others, thereby eliciting emotions in the viewer. These exploratory findings suggest that parents and caregivers might not only pay attention to the length of time that their children spend on YouTube but also to which channels and influencers they gravitate toward and why. Instead of making assumptions that YouTube consumption is contrary to educational or sleep goals, parents might investigate whether YouTube is a distraction or a coping mechanism or if it helps or hinders homework multitasking or sleep regulation. Educators and parents could partner in raising awareness of the multiple functions of YouTube in the home and at school, in particular how it can affect the readiness to learn, social–emotional learning, and attention.

## Figures and Tables

**Table 1:** Background details on YouTube channels mentioned by youth participants.

YouTube Channel Name	Creator Profile	Channel Description (from 2020)	Frequency of Posting	Example Quotes
PewDiePie (https://www.youtube.com/user/pewdiepie, accessed 15 September 2020, same for the rest links)	33-year-old male, Sweden	Gaming vlogs, reactions, commentary on pop culture	A few times a week	Lauren, 8th grade: I guess a star [that she watches] would be PewDiePie. He’s not Minecraft but he’s very popular. He’s the number one subscribed channel so I would hope he’s popular.
shane (https://www.youtube.com/user/shane)	34-year-old male, US	Conspiracies, reactions, vlogs, comedy sketches	Every month or less often	Lauren, 8th grade, when asked if she finds it difficult to sleep after watching YouTube: It depends on what I’m watching, like Shane Dawson, if you know who he is, sometimes I’ll be scared to go to sleep after, just like if he does the conspiracy type theories. Dimitry, 7th grade: He’s really funny. He wants things to explode, and things don’t. And it makes him mad and it’s really funny.
MrBeast (https://www.youtube.com/user/MrBeast6000)	24-year-old male, US	Challenges	Every other week	Chad H., 8th grade: They’re very entertaining because he always makes them entertaining and does random things that no one would ever do, and he puts them to e work I guess. Like real-life Battleship or something. Carlos, 8th grade: He’s a YouTuber that puts on videos or like “a last one to leave [from] playing gets $10,000”
emma chamberlain (https://www.youtube.com/channel/UC78cxCAcp7JfQPgKxYdyGrg)	21-year-old female, US	Lifestyle, vlogs, challenges	Every week	Catherine, 7th grade: She’s really funny and she doesn’t really care, like she’ll just go out with no makeup and a messy bun, and her videos are so relatable, and she is really funny.
Dolan Twins (https://www.youtube.com/user/TheDolanTwins/featured)	22-year-old males, US	Vlogs, challenges	Every other week	Lizzie, 8th grade: I like looking at all the nice stuff they have, their homes, like all the places they can go.
David Dobrik (https://www.youtube.com/@DavidDobrik)	26-year-old male, US	Vlogs with friends, challenges	Every other week	Lauren, 8th grade: He does crazy, kind of prank stuff, but he also does, surprises. Hismost common things he’s known for is like surprising his friends with cars. Catherine, 7th grade: David Dobrik—he isn’t the best role model, so I don’t watch him as often as I do of the sister squad…but they all do swear which I don’t really like. And it’s kind of annoying.
The ACE Family (https://www.youtube.com/@TheACEFamily)	30-year-old male and 32-year-old female, US, with children aged 6, 4, and 2	Family vlogs	A few times a week	Jenna, 6th grade: When I first heard about it, it was a family channel that lost their brother because of his heart. When I watch one of their videos, they always talk about him like a prayer. They say positive things that their brother said.

## Data Availability

Due to the in-depth, qualitative nature of the sources, the data are unavailable due to privacy and ethical restrictions.
